# Prognostic value of blood-based biomarkers in multiple sclerosis patients in the absence of clinical relapses or new MRI lesions

**DOI:** 10.1177/17562864251374903

**Published:** 2025-11-21

**Authors:** Tobias Brummer, Gabriel Gonzalez-Escamilla, Falk Steffen, Jasmin Jakob, Luisa Beyreuther, Sergiu Groppa, Stefan Bittner, Frauke Zipp, Vinzenz Fleischer

**Affiliations:** Department of Neurology, Research Center for Immunotherapy and Focus Program Translational Neuroscience, Rhine Main Neuroscience Network (rmn^2^), University Medical Center of the Johannes Gutenberg University Mainz, Mainz, Germany; Department of Neurology, Research Center for Immunotherapy and Focus Program Translational Neuroscience, Rhine Main Neuroscience Network (rmn^2^), University Medical Center of the Johannes Gutenberg University Mainz, Mainz, Germany; Department of Neurology, Research Center for Immunotherapy and Focus Program Translational Neuroscience, Rhine Main Neuroscience Network (rmn^2^), University Medical Center of the Johannes Gutenberg University Mainz, Mainz, Germany; Department of Neurology, Research Center for Immunotherapy and Focus Program Translational Neuroscience, Rhine Main Neuroscience Network (rmn^2^), University Medical Center of the Johannes Gutenberg University Mainz, Mainz, Germany; Department of Neurology, Research Center for Immunotherapy and Focus Program Translational Neuroscience, Rhine Main Neuroscience Network (rmn^2^), University Medical Center of the Johannes Gutenberg University Mainz, Mainz, Germany; Department of Neurology, Research Center for Immunotherapy and Focus Program Translational Neuroscience, Rhine Main Neuroscience Network (rmn^2^), University Medical Center of the Johannes Gutenberg University Mainz, Mainz, Germany; Department of Neurology, Research Center for Immunotherapy and Focus Program Translational Neuroscience, Rhine Main Neuroscience Network (rmn^2^), University Medical Center of the Johannes Gutenberg University Mainz, Mainz, Germany; Department of Neurology, Research Center for Immunotherapy and Focus Program Translational Neuroscience, Rhine Main Neuroscience Network (rmn^2^), University Medical Center of the Johannes Gutenberg University Mainz, Mainz, Germany; Department of Neurology, Focus Program Translational Neuroscience, Rhine Main Neuroscience Network (rmn^2^), University Medical Center of the Johannes Gutenberg University Mainz, Langenbeckstr. 1, Mainz 55131, Germany

**Keywords:** lesion volume, MRI, multiple sclerosis, neurofilament light, serum glial fibrillary acidic protein

## Abstract

**Background:**

In multiple sclerosis (MS), an increase in whole-brain lesion volume (LV) on MRI can be observed even in the absence of newly demarcated focal lesions or clinical relapses. However, it is unknown whether the presence of increasing LV alone is enough to justify changes in the therapeutic regimen. At this point, blood-based biomarkers may aid to identify patients at risk for progression.

**Objective:**

To determine the prognostic value of blood-based biomarkers (serum neurofilament (sNfL) and serum glial fibrillary acidic protein (sGFAP)) on disability progression in MS patients without newly demarcated lesions or clinical relapses.

**Design:**

Longitudinal cohort study.

**Methods:**

In total, out of 291 MS patients who were retrospectively screened for this study, 171 patients underwent a detailed clinical and MRI assessment and were finally included in the analysis: 100 patients with increasing LV (mean baseline Expanded Disability Status Scale (EDSS) = 1.5) and 71 with stable LV over 2 years (mean baseline EDSS = 1.0). Baseline blood-based measures (sNfL and sGFAP) and MRI metrics (total T2-weighted LV, gray matter (GM) volume) were acquired. EDSS worsening served as a clinical outcome measure and was determined through a 2-year follow-up. Receiver operator characteristic analyses were conducted to determine the predictive discriminative power of both blood-based biomarkers. Multivariate logistic regressions were performed to identify independent risk factors for EDSS progression in both cohorts.

**Results:**

MS patients with increasing LV had lower GM volume (*p* = 0.0109, *q* = 0.0490) and worse EDSS scores (*p* = 0.0065, *q* = 0.0650) at clinical follow-up compared to patients with stable LV. Patients with increasing LV and EDSS progression had significantly higher sNfL (*p* = 0.0049, *q* = 0.0196), but not sGFAP (*p* = 0.7425, *q* = 0.9900) levels. In the logistic regression model, sNfL levels remained an independent risk factor for EDSS progression in patients with increasing LV (odds ratio = 1.344, 95% confidence interval: 1.038–1.739, *p* = 0.025), but not in patients with stable LV. Finally, in patients with increasing LV, sNfL levels, but not sGFAP levels, discriminated progressive from non-progressive MS patients upon clinical follow-up (area under the curve = 0.67, *p* = 0.004; *q* = 0.016).

**Conclusion:**

sNfL enhances the prediction of disease progression in MS patients with merely increasing LV on MRI but no new T2 lesions or other signs of inflammatory activity. These findings may support treatment decisions in seemingly stable patients.

## Plain language summary

How blood tests can help predict disease progression in multiple sclerosis, even without new symptoms or new lesions

In multiple sclerosis (MS), an increase in lesion volume (LV) on MRI can be observed even in the absence of new lesions or clinical relapses. However, it is unknown whether the presence of increasing LV alone is enough to justify changes in the treatment. At this point, blood biomarkers may aid to identify patients at risk for progression. This study looked at whether certain blood tests could help to predict disease worsening in MS patients who appear stable, meaning they have no new symptoms or new lesions on MRI but do show an overall increase in the volume of lesions. Researchers studied 171 people with MS over a two-year period. Some had increasing lesion volume (LV) in the brain, while others had stable LV. They measured two proteins in the blood, namely neurofilament light chain (sNfL) and glial fibrillary acidic protein (sGFAP), which indicate damage to the brain. The authors found that patients with increasing LV but no new lesions over two years and higher sNfL, though not sGFAP, levels faced a higher risk of disability progression. Notably, sNfL, but not sGFAP, emerged as an independent risk factor for disability accumulation in this cohort. Thus, sNfL measurements can help to identify relapse-free patients without new lesions but increasing LV who are at risk of progression. This could help doctors to make better treatment decisions for patients who do not have clear signs of disease activity.

## Introduction

In multiple sclerosis (MS), disability progression is closely related to neuroaxonal degeneration.^1,2^ From the clinical point of view, disability accumulation can be acquired via relapse-associated worsening or progression independent of relapse activity (PIRA). However, the pathophysiology of the relapse-free accumulation of neurological disability driving insidious progression without overt relapses remains poorly understood. Subclinical disease activity is commonly monitored by MRI-based measures, such as new T2-hyperintense lesions, T1-hypointense lesions (known as black holes), or contrast-enhancing lesions. However, these metrics do not align well with disability progression.^3–6^ This mismatch, called the clinical-radiological paradox, has been extensively studied.^7^ The variability in lesion formation, growth rates, and location may explain part of this paradox.^4^ Hence, focusing on whole-brain T2-hyperintense lesion volume (LV) rather than simple lesion count has been suggested for tracking disability progression.^6^

Against this background, MS patients without new T2-hyperintense lesions in a subsequent MRI can show subtle radiological progression by demonstrating an increase in LV. Therefore, MS patients without new T2-hyperintense lesions can be further differentiated into those with increasing whole-brain LV and those without an increase.

Currently, it is unknown whether the presence of increased LV on MRI scans alone—without clinical relapses or new demarcated lesions—is enough to justify changes in the therapeutic regimen. Therefore, identifying individual patients with an increase in whole-brain lesion load who are also at risk of disease progression may require additional biomarkers. Despite the excellent spatial resolution of MRI measures, their prognostic ability may still be increased through combination with fluid biomarkers of neuroinflammation.

Recent technical advances, such as Single Molecule Array (SiMoA^®^), have enabled non-invasive measurements of neuroinflammatory- and neurodegeneration-related biomarkers in serum and plasma.^8,9^ Therefore, blood-based biomarkers such as serum neurofilament light chain (sNfL) and serum glial fibrillary acidic protein (sGFAP) have gained significant interest and are on the cusp of being applied in clinical routine diagnostics.^8,9^ Overall, sNfL has been shown to be a marker of acute inflammation,^10^ but also disease progression,^11^ while sGFAP has been primarily linked to PIRA.^12,13^ Together, these biomarkers may offer additional predictive value, particularly in patients at risk of progression who solely show an increase in overall lesion load in the absence of other signs of disease activity.

Here, we present a systematic longitudinal study to determine the predictive value of blood-based biomarkers (sNfL and sGFAP) for disability progression in MS patients with solely increasing whole-brain LV on MRI.

## Methods

### Participants

In total, 291 early MS patients (disease duration <98 years) underwent a comprehensive and detailed assessment, including baseline and follow-up MRI at the outpatient clinic of the Department of Neurology, at the University Medical Center Mainz (Germany; Table 1). The systematic longitudinal cohort study was approved by the local ethics committee; written informed consent was obtained from all patients. Inclusion criteria were a diagnosis of clinically isolated syndrome or relapsing-remitting MS (RMSS) according to the 2017 McDonald criteria, availability of high-quality MRI at both baseline and follow-up, and complete clinical data for the observation period (24 months). Exclusion criteria included clinical relapses or new T2 lesions during follow-up and incomplete imaging or laboratory data.

**Table 1.** Demographics and clinical characteristics.

Demographics	Increasing LV (*n* = 100)	Stable LV (*n* = 71)	*p*-Value	95% CI	*q*-Value
Age (years) mean ± SD (median, 25% and 75% perc.)	37 ± 10 (36; 28; 44)	32 ± 10 (32; 24; 41)	***0.0088***	−7.083 to −1.038	**0.0440**
Sex (female) (%)	76 (76.0)	50 (70.4)	*0.3784*	–	0.5410
Disease course					
RRMS (%)	80 (80.0)	58 (82.0)	*0.8458*	–	0.8458
CIS (%)	20 (20.0)	13 (18.0)			
Disease duration (years) mean ± SD (median, 25% and 75% perc.)	2.6 ± 4.6 (1.0; 0.0; 2.0)	2.1 ± 3.8 (1.0; 0.0; 3.0)	*0.4560*	−*1.768* to *0.7974*	0.5666
Disease-modifying treatment					
None (%)	26 (26.0)	17 (24.0)	*0.4533*	–	0.5067
Moderate (%)	55 (55.0)	45 (63.0)			
High (%)	19 (19.0)	9 (13.0)			
Clinical parameters					
Baseline EDSS mean ± SD (median, 25% and 75% perc.)	1.5 ± 1.5 (1.5; 0.0; 2.0)	1.2 ± 1.3 (1.0; 0.0; 2.0)	*0.0972*	−0.500 to 0.000	0.1620
Follow-up EDSS mean ± SD (median, 25% and 75% perc.)	1.9 ± 1.8 (2.0; 0.0; 3.0)	1.3 ± 1.3 (1.0; 0.0; 2.0)	***0.0065***	−1.000 to 0.000	0.0650
EDSS difference mean ± SD (median, 25% and 75% perc.)	0.4 ± 0.9 (0.0; 0.0; 0.5)	0.1 ± 0.5 (0.0; 0.0; 0.0)	***0.0102***	0.000–0.000	**0.0340**
Patients with EDSS progression (%)	36 (36.0)	14 (19.7)	***0.0267***	–	0.0668
Follow-up (months) mean ± SD (median, 25% and 75% perc.)	30 ± 18 (29; 15; 47)	26 ± 18 (23; 11; 42)	*0.0564*	−11.00 to 0.000	0.1128

Moderate = interferons, glatiramer acetate, teriflunomide, dimethyl fumarate; High = natalizumab, anti-CD20 monoclonal antibodies, sphingosine-1-phosphate receptor modulators, alemtuzumab, cladribine. Statistical significance is reported as *p*-values (italic; significant results are shown in bold), 95% CIs, and, where applicable, *q*-values after FDR correction.

CI, confidence interval; CIS, clinically isolated syndrome; EDSS, expanded disability status scale; FDR, false discovery rate; LV, lesion volume; RRMS, relapsing-remitting multiple sclerosis.

**Figure 1.**
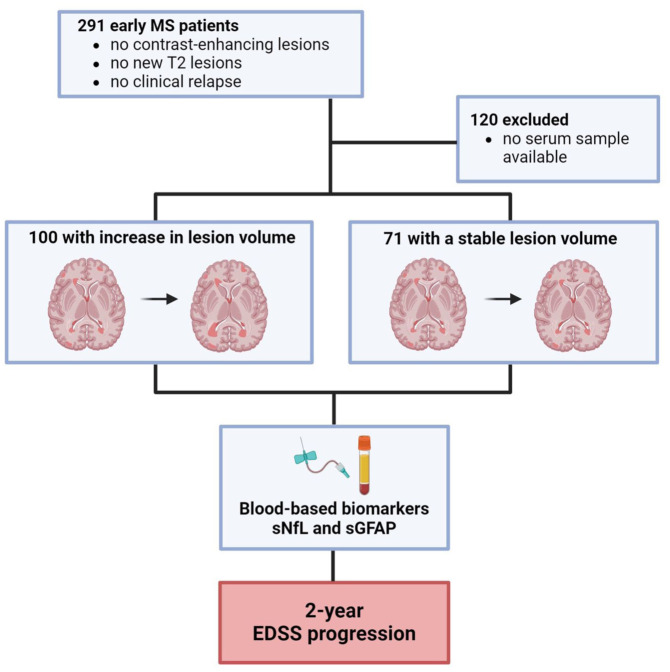
Study design and measurements. Illustration of the workflow diagram of our study cohort. EDSS, expanded disability status scale; MS, multiple sclerosis; sGFAP, serum glial fibrillary acidic protein; sNfL, serum neurofilament light chains.

The initial MS cohort (*n* = 291) comprised patients with no contrast-enhancing lesions at baseline and follow-up, no new T2 lesions, and no clinical relapses during the observation period of 24 months. From this selected MRI cohort, 120 patients were excluded, as there was no serum sample available. The remaining 171 patients were grouped into a cohort of patients with an increase in T2 LV (*n* = 100) and a cohort without an increase (*n* = 71; Figure 1). Of the 100 patients with increasing LV, 20 had clinically isolated syndrome (CIS) with no dissemination in time, whereas the remaining 80 had RRMS as diagnosed according to the 2017 revised McDonald diagnostic criteria.^14^ Of the 71 patients with stable LV, 13 had CIS, and 58 had a relapsing-remitting disease course. The median disease duration of all patients at study inclusion was 1.0 year for both groups. Blood-based biomarkers were measured from serum samples as described below. Blood and MRI baseline measurements were performed within 6 months of study inclusion. An experienced neurologist clinically assessed patients, including their Expanded Disability Status Scale (EDSS) score at study entry and follow-up visit (mean time to follow-up 30 months (increasing LV) and 26 months (stable LV)), along with demographic data. All measurements were performed at least 30 days after a high-dose corticosteroid treatment.

### SNfL and GFAP measurements

Serum samples were collected by attending physicians at the University Medical Center Mainz. Samples were processed at room temperature within 2 h. Serum samples were spun at 2000 × *g* at room temperature for 10 min, aliquoted in polypropylene tubes, and stored at −80°C. sNfL and sGFAP concentrations were measured using the highly sensitive SiMoA technology as previously described.^10,15,16^ Samples were measured in duplicates by SiMoA HD-1 (sNfL) and HD-X (sGFAP; Quanterix, Billercia, MA, USA) using NF-Light Advantage and GFAP Discovery kits according to the manufacturer’s instructions. The mean inter-assay and intra-assay coefficient of variation was less than 10%. Measurements were performed in a blinded fashion without information about clinical data. sNfL levels were transformed to *z*-scores adjusted for age and body mass index.^13^

### MRI data acquisition

MRI data acquisition was performed as previously described.^15^ In brief, structural MRI was acquired on a 3-Tesla MRI scanner (Magnetom Tim Trio, Siemens, Germany) with a 32-channel receive-only head coil. In all patients, imaging was performed using a sagittal 3D T1-weighted magnetization-prepared rapid gradient echo sequence (TE/TI/TR = 2.52/900/1900 ms, flip angle = 9°, field of view = 256 × 256 mm^2^, matrix size = 256 × 256, slab thickness = 192 mm, voxel size = 1 × 1 × 1 mm^3^) and a sagittal 3D T2-weighted fluid-attenuated inversion recovery (FLAIR) sequence (TE/TI/TR = 388/1800/5000 ms, echo-train length = 848, field of view = 256 × 256 mm^2^, matrix size = 256 × 256, slab thickness = 192 mm, voxel size = 1 × 1 × 1 mm^3^). A clinician scientist blinded to the patient data excluded major anatomical abnormalities based on the subject’s T1-weighted and FLAIR images of the whole brain.

### Quantification of white matter lesion, gray matter volume, and increasing LV

The quantification of white matter (WM) lesions and gray matter (GM) volume was performed as previously described.^15^ In brief, the volumes of WM lesions were evaluated using the cross-sectional pipeline of the lesion segmentation toolbox,^17^ under the Statistical Parametric Mapping (SPM8) software. 3D FLAIR images were co-registered to 3D T1-weighted images and bias corrected. After partial volume estimation, lesion segmentation was performed with 20 different initial threshold values for the lesion growth algorithm.^17^ For each patient, the optimal threshold (*k* value, dependent on image contrast) was determined, and average values were calculated. A uniform *k* value of 0.1 was applied in all patients to automatically estimate LV and filling of 3D T1-weighted images. Subsequently, the filled 3D T1-weighted images and the native 3D T1-weighted images were segmented into GM, WM, and cerebrospinal fluid (CSF) and then normalized to Montreal Neurological Institute space. The quality of the segmentations was visually inspected to increase reliability. Volumes were further normalized by total brain volume (brain parenchymal volume plus CSF volume) to calculate the brain parenchymal fraction (BPF) and GM fraction (GMF), thereby accounting for differences in head and brain size. Patients were classified as having increasing T2 LV when they did not have new T2 lesions but showed an overall increase in whole-brain T2 LV on follow-up MRIs. Patients who did not have new T2 lesions but showed a stable or reduced T2 LV on follow-up MRIs were grouped as patients with stable LV.

### Statistical analysis

Statistical analysis was performed using SPSS 23 (SPSS, Chicago, IL, USA), MedCalc (Version 20.115, Medcalc Software Ltd., Ostend, Belgium), and GraphPad Prism 9 software. Summary statistics are presented as mean, standard deviation, median (25th and 75th percentile), or number (percentage), where applicable. Furthermore, predictive discriminating values were calculated by a receiver operating characteristic (ROC) analysis using MedCalc. This statistical method is preferentially used to make a series of discriminations into two different states (e.g., EDSS progression vs no EDSS progression) based on a specific diagnostic variable (e.g., sNfL). Every value of that discriminating variable is used as a cut-off with the calculation of the corresponding sensitivity and specificity. Normal distribution was evaluated by the Kolmogorov-Smirnov and Shapiro-Wilk tests. Variables that did not pass normality tests underwent Mann-Whitney tests. Normally distributed data underwent *t* tests. Chi-square test of homogeneity was used to compare differences in proportions. ANOVA was used to compare group means across multiple variables. *p*-Values less than 0.05 were considered statistically significant. All *p*-values were corrected for multiple comparisons using the Benjamini-Hochberg false discovery rate (FDR) procedure. Corrected *p*-values are reported as *q*-values.

## Results

### Patient characteristics

We included 291 early MS patients whose MRI did not show any new contrast-enhancing T1 lesions, T2 lesions, or clinical relapses over a 2-year monitoring period (Figure 1). 120 patients were excluded from the final analysis because there were no serum samples available. Of the remaining patients, 100 were categorized as patients with increasing LV and 71 as patients with stable LV, as outlined in the “Methods” section. All demographics and clinical characteristics of the investigated cohorts are summarized in Table 1. The mean time to follow-up for both groups was 30 ± 18 months (increasing LV) and 26 ± 18 months (stable LV), respectively (*p* = 0.0564, *q* = 0.1128). The disease duration of both cohorts was comparable (mean: 2.6 ± 4.6 vs 2.1 ± 3.8 years; *p* = 0.4560, *q* = 0.5666), as were baseline disease-modifying treatments (DMTs; *p* = 0.4533, *q* = 0.5067). The majority of patients had RRMS (80.0% vs 82.0%), and this distribution was similar between groups (*p* = 0.8458, *q* = 0.8458).

Notably, patients with increasing LV were significantly older than those with stable LV (mean age: 37 ± 10 vs 32 ± 10 years; *p* = 0.0088), and this difference remained significant after FDR correction (*q* = 0.0440). The sex distribution was not significantly different (76.0% vs 70.4% females; *p* = 0.3784, *q* = 0.5410). Baseline EDSS scores were also comparable (mean: 1.5 ± 1.5 vs 1.2 ± 1.3; *p* = 0.0972, *q* = 0.1620).

However, patients with increasing LV had higher EDSS scores at follow-up (mean: 1.9 ± 1.8 vs 1.3 ± 1.3; *p* = 0.0065), though this difference did not reach significance after FDR correction (*q* = 0.0650). The mean change in EDSS was also greater in the increasing LV group (0.4 ± 0.9 vs 0.1 ± 0.5; *p* = 0.0102), which remained significant after FDR correction (*q* = 0.0340). Furthermore, a greater proportion of patients with increasing LV experienced EDSS progression compared to those with stable LV (36.0% vs 19.7%; *p* = 0.0267), although this difference did not remain significant after correction (*q* = 0.0668).

### Patients with increasing LV showed an elevation of sNfL and sGFAP

Subsequently, we explored whether these differences in clinical outcome measures could be attributed to disparities in blood- and imaging-based biomarkers between the two groups. Overall, sNfL levels tended to be slightly higher in patients with high-efficacy DMTs; however, this was not statistically significant (means: 0.27 ± 1.94 (none); 0.37 ± 1.94 (moderate efficacy); and 1.22 ± 1.95 (high efficacy); *p* = 0.0912).

**Figure 2.**
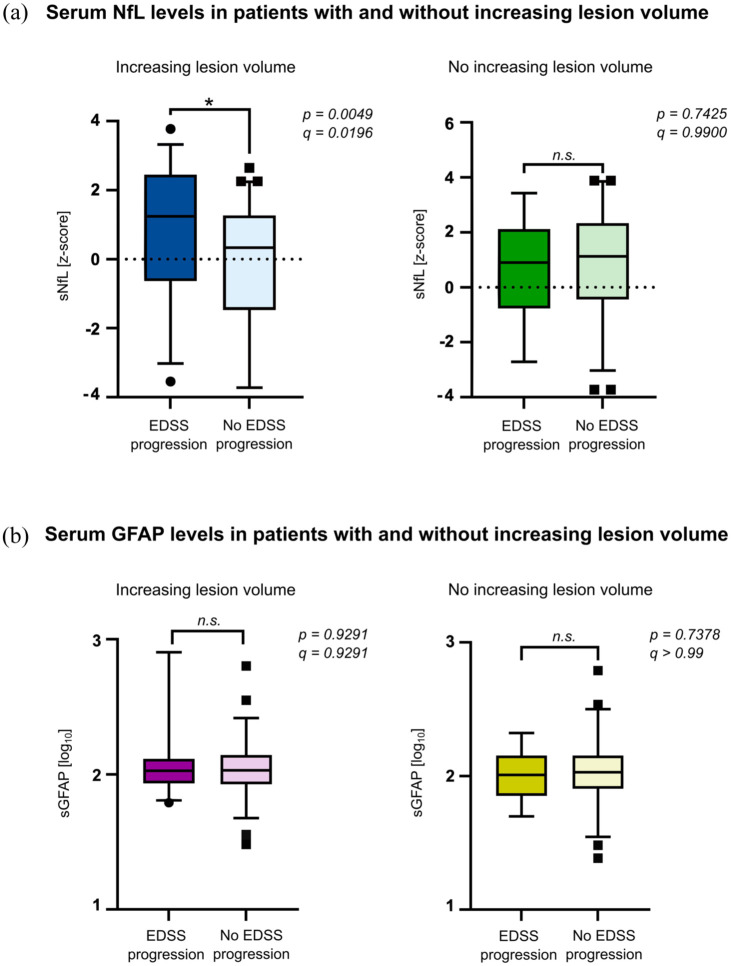
Blood-based biomarkers in patients with increasing and stable LV. (a) Mean sNfL *z*-scores in MS patients with increasing and stable LV. Statistical significance is reported as *q*-values after FDR correction. (b) Mean sGFAP levels (log_10_ transformed) in patients with increasing and stable LV. Statistical significance is reported as *q*-values after FDR correction. EDSS, expanded disability status scale; FDR, false discovery rate; LV, lesion volume; MS, multiple sclerosis; sGFAP, serum glial fibrillary acidic protein; sNfL, serum neurofilament light chains.

Patients with stable LV had higher sNfL levels (*z*-scores, mean: 0.85 ± 1.96 vs 0.21 ± 1.93; *p* = 0.0387), although this difference did not remain significant after FDR correction (*q* = 0.0871; Table 2). However, patients who exhibited EDSS progression did not show a significant difference in sNfL levels when compared to those without EDSS progression in this group (mean: 0.696 ± 1.97 vs 0.892 ± 1.97; *p* = 0.7425; *q* = 0.9900; Figure 2(a), right panel). Conversely, patients with increasing LV and EDSS progression had significantly higher sNfL levels than patients with increasing LV and no EDSS progression (mean: 0.95 ± 1.86 vs −0.174 ± 1.86; *p* = 0.0049; *q* = 0.0196; Figure 2(a), left panel). Notably, sGFAP levels did not exhibit significant differences between the two groups (mean (log_10_ sGFAP): 2.07 and 2.04 *p* = 0.9291, *q* = 0.9291 vs 2.01 and 2.03, *p* = 0.737, *q* > 0.99) regardless of EDSS progression (Figure 2(b)).

**Table 2.** Comparison of the blood-based biomarkers and the MRI-derived measures.

Blood- and imaging measures	Increasing LV (*n* = 100)	Stable LV (*n* = 71)	*p*-Value	95% CI	*q*-Value
sNfL (*z*-score) mean ± SD (median, 25% and 75% perc.)	0.21 ± 1.93 (0.52; −1.13; 1.65)	0.85 ± 1.96 (1.13; −0.58; 2.26)	***0.0387***	0.030–1.240	0.0871
sGFAP (pg/ml) mean ± SD (median, 25% and 75% perc.)	139 ± 187 (107; 85; 134)	125 ± 88 (107; 78; 143)	*0.8484*	−17.11 to 12.94	0.8484
Baseline LV (ml) mean ± SD (median, 25% and 75% perc.)	6.57 ± 12.3 (1.91; 0.82; 6.28)	5.55 ± 7.12 (2.05; 0.80; 6.79)	*0.6307*	−0.522 to 0.867	0.7095
Follow-up LV (ml) mean ± SD (median, 25% and 75% perc.)	7.65 ± 13.3 (2.51; 1.15; 7.80)	4.91 ± 6.63 (1.93; 0.67; 5.25)	*0.1174*	−1.435 to 0.111	0.1761
Delta LV (ml) mean ± SD (median, 25% and 75% perc.)	1.08 ± 1.45 (0.45; 0.15; 1.64)	−0.65 ± 1.06 (−0.28; −0.61; −0.11)	***<0.0001***	−1.503 to −0.789	**0.0009**
Baseline BPF mean ± SD (median, 25% and 75% perc.)	0.83 ± 0.03 (0.84; 0.82; 0.86)	0.84 ± 0.02 (0.84; 0.83; 0.85)	*0.1405*	−0.002 to 0.012	0.1806
Follow-up BPF mean ± SD (median, 25% and 75% perc.)	0.83 ± 0.03 (0.84; 0.82; 0.85)	0.84 ± 0.02 (0.84; 0.83; 0.86)	*0.0891*	−0.001 to 0.014	0.1604
Baseline GMF mean ± SD (median, 25% and 75% perc.)	0.43 ± 0.04 (0.44; 0.41; 0.46)	0.44 ± 0.03 (0.45; 0.43; 0.47)	***0.0209***	0.002–0.022	0.0627
Follow-up GMF mean ± SD (median, 25% and 75% perc.)	0.42 ± 0.04 (0.43; 0.40; 0.45)	0.44 ± 0.03 (0.44; 0.42; 0.46)	***0.0109***	0.004–0.025	**0.0490**

Statistical significance is reported as *p*-values (italic; significant results are shown in bold), 95% CI, and, where applicable, *q*-values after FDR correction.

BPF, brain parenchymal fraction; CI, confidence interval; FDR, false discovery rate; GMF, gray matter fraction; LV, lesion volume; sGFAP, serum glial fibrillary acidic protein; sNfL, serum neurofilament light chain.

Consistent with the results from the blood-based biomarkers, patients with increasing LV exhibited lower GM volume both at baseline (mean GMF: 0.43 ± 0.04 vs 0.44 ± 0.03; *p* = 0.0209), which showed a trend after FDR correction (*q* = 0.0627), and at follow-up (mean GMF: 0.42 ± 0.04 vs 0.44 ± 0.03; *p* = 0.0109), which remained significant after correction (*q* = 0.0490; Table 2). Baseline and follow-up BPF did not differ significantly between the groups (baseline BPF: mean 0.83 ± 0.03 vs 0.84 ± 0.02; *p* = 0.1405, *q* = 0.1806; follow-up BPF: mean 0.83 ± 0.03 vs 0.84 ± 0.02; *p* = 0.0891, *q* = 0.1604), though both showed a non-significant trend toward lower BPF in patients with increasing LV.

Baseline T2 LV were comparable between the two groups (mean: 6.57 ± 12.3 vs 5.55 ± 7.12 ml; *p* = 0.6307, *q* = 0.7095), and follow-up T2 LV displayed only a trend toward larger volumes in patients with increasing LV (mean: 7.65 ± 13.3 vs 4.91 ± 6.63 ml; *p* = 0.1174, *q* = 0.1761). In contrast, the change in lesion volume (ΔLV) between baseline and follow-up was highly significant (mean: 1.08 ± 1.45 vs −0.65 ± 1.06 ml; *p* < 0.0001) and remained robust after FDR correction (*q* = 0.0009).

Next, we constructed a multivariate logistic regression model that incorporated sNfL or sGFAP levels, sex, age, disease duration, DMT, and MRI variables (BPF and GMF) as covariates. Importantly, sNfL remained a significant independent predictor of EDSS progression (odds ratio (OR) = 1.344, 95% confidence interval (CI): 1.038–1.739, *p* = 0.025) in patients with increasing LV, whereas sGFAP levels (OR = 1.001; *p* = 0.525; 95% CI: 0.998–1.004) did not exhibit such an association (Table 3). Moreover, MRI variables did not significantly contribute to the prediction model. Given that age is already accounted for in sNfL *z*-scores,^13^ it was not included as an additional covariate in the multivariate regression models for sNfL.

**Table 3.** Multivariate logistic regression model.

EDSS progression (yes/no)	Odds ratio	*p*-Value	95%-CI
Increasing LV (*n* = 100)			
sNfL (*z*-score)	1.344	***0.025***	1.038–1.739
sGFAP (pg/ml)	1.001	*0.525*	0.998–1.004
Stable LV (*n* = 71)			
sNfL (*z*-score)	0.964	*0.828*	0.695–1.338
sGFAP (pg/ml)	0.999	*0.871*	0.989–1.009

Covariates for sNfL (*z*-score): sex, disease duration, DMT, GMF, BPF. Covariates for sGFAP: sex, age, disease duration, DMT, GMF, BPF. Statistical significance is reported as *p*-values (italic, significant results are shown in bold) and 95% CI.

BPF, brain parenchymal fraction; CI, confidence intervals; DMT, disease-modifying treatment; EDSS, expanded disability status scale; GMF, gray matter fraction; LV, lesion volume; sGFAP, serum glial fibrillary acidic protein; sNfL, serum neurofilament light chain.

### Predictive value of blood-based biomarkers in patients with increasing and stable LV

**Figure 3.**
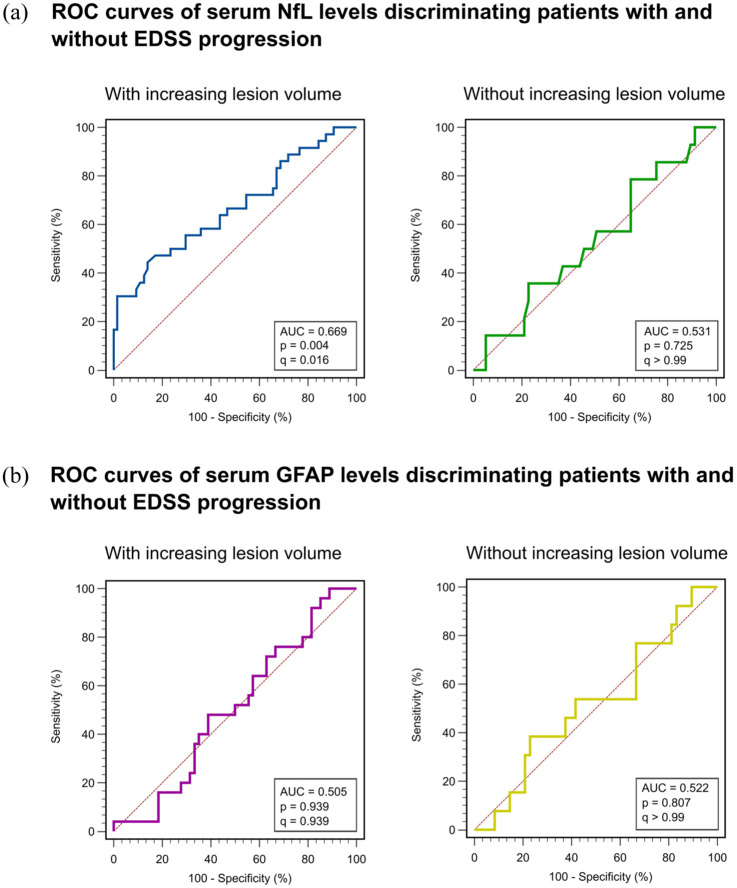
ROC curves. (a) ROC curves of sNfL levels classifying patients based on their EDSS progression state over 2 years in patients with increasing (left panel) and stable LV (right panel). Statistical significance is reported as *p*-values and *q*-values after FDR correction. (b) ROC curves of sGFAP levels classifying patients based on their EDSS progression over 2 years in patients with increasing (left panel) and stable LV (right panel). Statistical significance is reported as *p*-values and *q*-values after FDR correction. EDSS, expanded disability status scale; FDR, false discovery rate; LV, lesion volume; ROC, receiver operating characteristic; sGFAP, serum glial fibrillary acidic protein; sNfL, serum neurofilament light chains.

Finally, we aimed to determine the predictive discriminative potential of blood-based biomarkers for EDSS progression in patients with increasing and stable LV. In the ROC analysis, we found that baseline sNfL levels exhibited an area under the curve (AUC) of 0.669 (95% CI: 0.568–0.760, *p* = 0.004; *q* = 0.016) with a sensitivity of 44.4% and a specificity of 85.9% for patients with increasing LV. The Youden index, the maximum potential effectiveness of a biomarker, was 0.304 (95% CI: 0.1243–0.4188; Figure 3(a)). In patients with stable LV, sNfL levels only demonstrated an AUC of 0.531 (95% CI: 0.1178–0.1366, *p* = 0.7251; *q* > 0.990; Figure 3(b)). In contrast to sNfL, sGFAP levels did not possess the capability to predict EDSS progression in either group (Figure 3(b)). Notably, the additional inclusion of MRI-based neurodegenerative measures (GMF or BPF) did not substantially improve model performance (AUC = 0.681 and 0.672, respectively) compared to the model using sNfL alone (AUC = 0.669), underscoring the robust predictive value of sNfL independent of MRI-derived neurodegenerative measures (Figure S1).

## Discussion

In MS, early identification of patients at risk of disease progression is crucial for implementing timely therapeutic interventions. This study evaluated the prognostic value of sNfL and sGFAP levels in predicting progression in relapse-free MS patients without new MRI lesions. We found that patients with increasing LV but no new T2 lesions over 2 years and higher sNfL—though not sGFAP—levels faced a higher risk of disability progression. Notably, sNfL, but not sGFAP, emerged as an independent risk factor for disability accumulation in this cohort. Thus, sNfL measurements can help identify relapse-free patients without new lesions but with increasing LV who are at risk of disease progression.

In contrast to our results, there is some previous evidence highlighting sGFAP as a marker of disease progression, in particular PIRA.^12,18^ However, these studies primarily involved cohorts in more advanced disease stages, specifically patients with progressive MS.^19,20^ When investigating sGFAP as a marker for disease progression in cohorts with RRMS, prior studies have shown rather heterogeneous results.^12,21–23^ These differences may be attributed to the overall higher abundance of lesions with a larger content of astrocytes and glial scarring, such as inactive and remyelinating lesions in patients with progressive MS.^24–26^ In our study, the weaker performance of sGFAP as a prognostic marker may reflect the focus on a relapsing MS cohort with a relatively short 2-year follow-up, during which neuroaxonal damage—as reflected by sNfL—may align more closely with disease progression. Moreover, sGFAP may better capture astrocytic activation and gliosis evolving over longer periods and in progressive MS. However, these subtypes were not included in our study population. This underscores the need for future studies with longer follow-up and broader inclusion of progressive MS to fully evaluate the prognostic utility of sGFAP.

Overall, our results indicate that disease progression in early MS patients with increasing LV primarily relates to neuroaxonal degeneration (sNfL) rather than glial scar formation or astrocyte damage or activation (sGFAP). These results indeed support recently suggested models indicating neurodegeneration as a main driver, paralleling neuroinflammation, during the initial stages of MS.^27^

Here, our study demonstrates that sNfL measurements could offer added value in identifying individual patients with increasing LV, who are at risk of disease progression, even in the absence of clinical relapses or newly demarcated lesions. These results align well with previous histopathological and transcriptomic characterizations of enlarging lesions, revealing extensive neuroaxonal damage within lesion edges,^28^ along with a profound accumulation of proinflammatory microglia.^29,30^ Especially CNS myeloid cells, such as microglia, have been shown to play a pivotal role in mediating neuroaxonal damage and subsequent neurofilament release during neuroinflammation.^31^ Therefore, disease progression in patients with increasing LV may point toward the significance of smoldering microglial activation for progression-promoting pathology in MS. Hence, the growing armamentarium of disease-modifying therapies, such as Bruton’s tyrosine kinase inhibitors, may offer new therapeutic strategies to tackle such microglia-driven neuroinflammation and subsequent neuroaxonal damage.

In addition, our study also exemplifies that combining biomarkers from different modalities,^15^ such as longitudinal MRI (presence of increasing LV) and blood-based biomarkers (increased sNfL), may enhance accuracy in identifying a specific subset of patients at risk of disease progression.^32^ In a complex condition such as MS, the predictive power of one single biomarker is usually not sufficient to depict the intricate interplay of inflammation, degeneration, and repair. Hence, future diagnostic assessments of patients with MS may require the measurement of a subset of cross-modal biomarkers to offer a more comprehensive understanding of ongoing disease mechanisms.^32^

The increasing whole-brain T2 lesion load is likely due to enlarging lesions that are characterized by the gradual increase of demyelinated areas around the lesion core and primarily represent a hallmark of progressive MS.^33–35^ However, these lesions are also present in earlier disease stages.^36,37^ Accumulation of these chronic active lesions contributes to the maintenance of inflammation, chronic demyelination, and axonal loss, driving disability in MS,^38^ and hence it is essential to identify standardized in vivo surrogate markers. Although a standardized definition for chronic active lesions on MRI scans is yet to emerge, recent MRI advancements have identified two distinct imaging correlates: slowly expanding lesions (SELs) and paramagnetic rim lesions (PRLs).^39–41^ Histopathologically, these lesions feature a hypomyelinated core and, in the case of PRLs, a rim of iron-laden cells from myeloic and astrocytic lineages.^25,30,36^ Recently, advances in RNA sequencing have enabled the detection of specific accumulating microglial and astrocytic cell populations in paramagnetic rims of chronic active lesions, which may represent the cellular correlate of smoldering WM lesions.^30^

While acknowledging the significance of our findings, our study also has some limitations to be considered. First, we utilized a simplified definition of increasing lesion load using conventional, but also broadly available, MRI sequences, which may overlook individual lesion characteristics.^35,39^ Susceptibility-weighted imaging for PRLs and computation of deformation field maps for SELs would offer more information about the chronic active state of the lesions. Thus, processes within individual lesions (chronic activity, inflammation resolution, and tissue loss) may have hindered the predictive power of our study. However, our approach might also enhance its applicability in the broad routine clinical setting. In addition, our study was conducted on a real-world cohort. Therefore, not every biomarker was measured at the exact same time. However, a real-world cohort closely mirrors a more realistic clinical situation, potentially reducing the risk of selection bias.^42^ Third, despite the longitudinal MRI and clinical assessment, we had no follow-up biomarker data for our cohort. However, our study was primarily conceptualized to predict disease progression based on an initial set of biomarkers. Furthermore, it is important to emphasize that while ultrasensitive assays like SiMoA have markedly advanced the detection and application of blood-based biomarkers in MS, recent studies have underscored the necessity of cautious interpretation in clinical settings. Variability in assay performance, biological confounders, and the inherent complexity of MS pathophysiology all contribute to challenges in applying these biomarkers reliably.^43^ Regarding study design, a formal power analysis for sample size calculation was not conducted, as this retrospective observational study utilized all available real-world data, rendering pre-study power estimation inapplicable. Finally, disease progression could potentially be driven by spinal cord lesions; however, this is unlikely since the patients did not experience clinical relapse.

## Conclusion

In conclusion, our study highlights sNfL as a valuable biomarker for predicting disease progression in early MS patients with increased whole-brain T2 lesion load but no new T2 lesions or other signs of disease activity. These findings may support treatment decisions in this particular cohort of patients. Larger, more diverse studies are needed to confirm these results and establish the clinical utility of these biomarkers in guiding MS therapy.

## Supplemental Material

sj-pdf-1-tan-10.1177_17562864251374903 – Supplemental material for Prognostic value of blood-based biomarkers in multiple sclerosis patients in the absence of clinical relapses or new MRI lesions Supplemental material, sj-pdf-1-tan-10.1177_17562864251374903 for Prognostic value of blood-based biomarkers in multiple sclerosis patients in the absence of clinical relapses or new MRI lesions by Tobias Brummer, Gabriel Gonzalez-Escamilla, Falk Steffen, Jasmin Jakob, Luisa Beyreuther, Sergiu Groppa, Stefan Bittner, Frauke Zipp and Vinzenz Fleischer in Therapeutic Advances in Neurological Disorders

## Declarations

**Ethics approval and consent to participate:** The study was approved by the local ethics committee of the University Medical Center of the Johannes Gutenberg University Mainz (numbers: 837.363.10 (7372); 837.479.17 (11313), 2018-13622; 837.019.10 (7028)). Written informed consent was obtained from all patients.

**Consent for publication:** Written informed consent was obtained from all patients.

**Availability of data and materials:** Restrictions apply to the availability of these data, which were used under license for the current study and are therefore not publicly available. The raw data used in preparation of the figures and tables will be shared in an anonymized format upon reasonable request by a qualified investigator for purposes of replicating procedures and results.
