# Supplementary material for: Prognostic value of blood-based biomarkers in multiple sclerosis patients in the absence of clinical relapses or new MRI lesions
Source: Ther Adv Neurol Disord. 2025 Nov 21;18:17562864251374903. doi: 10.1177/17562864251374903 (PMC12639232; doi:10.1177/17562864251374903)
Supplement: sj-pdf-1-tan-10.1177_17562864251374903 – Supplemental material for Prognostic value of blood-based biomarkers in multiple sclerosis patients in the absence of clinical relapses or new MRI lesions [file sj-pdf-1-tan-10.1177_17562864251374903.pdf]

Supplementary Figure 1

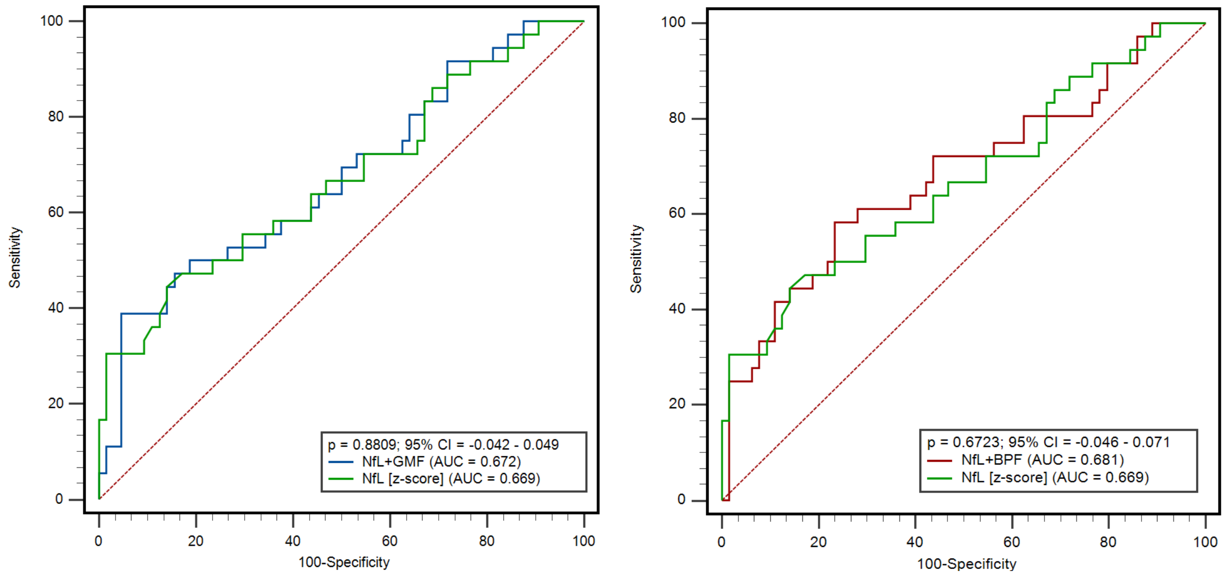

Supplementary Figure 1. Receiver operating characteristic (ROC) curves including sNfL and imaging biomarkers. ROC curves of sNfL levels and sNfL+imaging biomarkers (left panel: NfL+GMF; right panel: NfL+BPF) classifying patients based on their EDSS progression state over two years in patients with increasing LV. Statistical significance is reported as p-values.

**AUC**	Area under the curve
**BPF**	Brain parenchyma fraction
**EDSS**	Expanded disability status scale
**GMF**	Grey matter fraction
**sNfL**	Serum neurofilament light chains
**ROC**	Receiver Operating Characteristic
